# Systematic review and meta‐analysis of internet‐delivered interventions providing personalized feedback for weight loss in overweight and obese adults

**DOI:** 10.1111/obr.12396

**Published:** 2016-03-07

**Authors:** A. Sherrington, J. J Newham, R. Bell, A. Adamson, E. McColl, V. Araujo‐Soares

**Affiliations:** ^1^ Institute of Health and SocietyNewcastle UniversityNewcastle upon TyneUK; ^2^ Human Nutrition Research CentreNewcastle UniversityNewcastle upon TyneUK; ^3^ Fuse–UKCRC Centre for Translational Research in Public HealthNewcastle UniversityNewcastle upon TyneUK; ^4^ Newcastle Clinical Trials UnitNewcastle UniversityNewcastle upon TyneUK

**Keywords:** Feedback, internet interventions, obesity, weight loss

## Abstract

**Background:**

Obesity levels continue to rise annually. Face‐to‐face weight loss consultations have previously identified mixed effectiveness and face high demand with limited resources. Therefore, alternative interventions, such as internet‐delivered interventions, warrant further investigation. The aim was to assess whether internet‐delivered weight loss interventions providing personalized feedback were more effective for weight loss in overweight and obese adults in comparison with control groups receiving no personalized feedback.

**Method:**

Nine databases were searched, and 12 studies were identified that met all inclusion criteria.

**Results:**

Meta‐analysis, identified participants receiving personalized feedback via internet‐delivered interventions, had 2.13 kg mean difference (SMD) greater weight loss (and BMI change, waist circumference change and 5% weight loss) in comparison with control groups providing no personalized feedback. This was also true for results at 3 and 6‐month time points but not for studies where interventions lasted ≥12 months.

**Conclusion:**

This suggests that personalized feedback may be an important behaviour change technique (BCT) to incorporate within internet‐delivered weight loss interventions. However, meta‐analysis results revealed no differences between internet‐delivered weight loss interventions with personalized feedback and control interventions ≥12 months. Further investigation into longer term internet‐delivered interventions is required to examine how weight loss could be maintained. Future research examining which BCTs are most effective for internet‐delivered weight loss interventions is suggested.

## Introduction

Obesity is of growing concern owing to the rise in prevalence with levels reaching 26% in men and 24% in women within the UK 1. In 2013, 83% of UK households had access to the internet, the vast majority through broadband connections, with over half of users able to connect to the internet via their mobile phones 2. Globally, the internet is accessed by over three billion people, over 40% of the world population 3.

Traditional weight loss interventions, such as in‐person consultations, have reported mixed findings for effectiveness in terms of weight loss and its sustainability 4, 5, which may be related to poor adherence rates. Reasons for non‐adherence within in‐person consultations include personal reasons, cost of travel, limited availability and lack of parking at venues 6. Internet‐delivered weight loss interventions could minimize these problems by increasing the convenience and control for the user and health professional and reducing the cost of an intervention 7, 8. The number of studies incorporating internet‐delivered weight loss interventions has increased over recent years 9. Previous reviews have demonstrated that internet‐delivered weight loss interventions can be effective in promoting weight loss and changes in physical activity and diet 10, 11, 12, 13. However, several reviews have shown heterogeneity in results between studies, with several reporting no consistent benefits of internet‐delivered weight loss interventions in comparison with control groups 10, 11, 14, 15, 16. Furthermore, many studies have demonstrated high attrition rates for both intervention and control groups 10, 13, 17.

Previous reviews have identified the need to identify which components of internet interventions contribute to weight loss and the effectiveness of an intervention. Taxonomies have been developed to provide definitions of active ingredients within interventions based on pre‐established descriptions of behaviour change techniques (BCTs) and how these relate to theories 18. Using these taxonomies allows researchers to identify the presence of BCTs within an intervention and promotes consistent reporting whilst enabling comparison and replication of intervention features 19.

Feedback has been identified as an important and effective component within technology‐based weight loss interventions 20, 21, 22. Feedback delivered by a person as part of an internet‐delivered intervention can encourage, motivate and assist patients in successfully completing a weight loss program 23. Control theory 24 incorporates the BCT of ‘providing feedback’. The theory's basic construct is known as the discrepancy‐reducing feedback loop. This process is considered to be key to self‐regulation. Self‐regulation processes can be used to reduce the intention‐behaviour gap and facilitate the understanding of the progression from intention to action. Self‐regulation‐based interventions have been identified as twice as effective as interventions without self‐regulation strategies 25. The use of internet‐delivered interventions can enhance weight loss effectiveness when individualized feedback and email counselling are integrated 21. Personalized feedback is generally delivered via specific tailored contacts, either web‐based messaging, emails, short message service or in‐person 26. It is important to identify and evaluate the types of feedback, which can be delivered via the internet.

The aim of the current study was to assess whether internet‐delivered weight loss interventions providing personalized feedback (IWLPF) were more effective for weight loss in overweight and obese adults in comparison with control groups either placed on a wait list, receiving a minimal face‐to‐face intervention or receiving internet‐delivered weight loss interventions without personalized feedback. In addition, it aims to describe how feedback is provided and to identify the BCTs incorporated within internet‐delivered weight loss interventions.

## Method

Guidelines set out in the Cochrane handbook for systematic reviews of interventions were followed 27, and reporting is in accordance with the PRISMA statement checklist 28. The review proposal was accepted onto PROSPERO (international prospective register for systematic reviews) on 17 May 2012, registration number: CRD42012002115.

### Search methods for identification of studies

#### Electronic databases

Databases searched were Scopus (1960‐present), Web of Science (1970‐present), EMBASE (1974‐present), MEDLINE (1948‐present), PsycINFO (1967‐present), ASSIA (1987‐present), IBSS (1951‐present), the Sociological Abstracts (1952‐present), CINAHL (1981‐present) and Clinical Trial registers (ISRCTN registry, EU Clinical Trials registry, WHO International Clinical Trials registry platform).

#### Search strategy

Databases were searched with combinations of the key words ‘internet’, ‘web’, ‘computer’, ‘online’, ‘eHealth’, ‘nutrition’, ‘diet*’, ‘physical activity’, ‘exercise’, ‘weight’, ‘weight loss’, ‘overweight’, ‘obes*’, ‘randomi*ed controlled trial’, ‘randomi*ed’, ‘randomi*ed trial’, ‘randomi*ed clinical’, ‘controlled clinical trial’ and ‘clinical trial’.

#### Inclusion criteria

Criteria for considering studies are outlined in Table 1. The definition used to code for the BCT feedback was taken from the CALO‐RE taxonomy definition of “Provide feedback on performance ‐ This involves providing the participant with data about their own recorded behaviour or commenting on a person's behavioural performance (e.g. identifying a discrepancy between behavioural performance and a set goal or a discrepancy between one's own performance in relation to others).” pg. 9 29. This definition was used throughout to guide the selection and inclusion process, coding and analysis. Reference lists of identified studies and citation indexes of papers citing the identified studies were searched. Relevant authors in the field were contacted and asked if aware of any other studies relevant to the review.

**Table 1:** Inclusion criteria to select studies for the systematic review

Inclusion criteria	
Population	Adult (18+ years) participants with BMI > 25 kg/m^2^
Interventions	Targeting diet and/or physical activity for weight loss
	Delivered at least in part via the internet
	Incorporating any form of individualized feedback to the participants either human‐delivered (provided by a health care professional or researcher) or computer‐generated personalized feedback (using algorithms that sent pre‐programmed responses based on participant input or choices) delivered via web‐based messages or email
	Definition of feedback used to guide process “Provide feedback on performance ‐ This involves providing the participant with data about their own recorded behaviour or commenting on a person's behavioural performance (e.g. identifying a discrepancy between behavioural performance and a set goal or a discrepancy between one's own performance in relation to others).” pg. 9 29
Comparator	Arms comprising no individualized feedback, e.g. wait list, treatment‐as‐usual, intervention without feedback
Outcome	Primary: body weight change
	Secondary: body fat, waist circumference or BMI change, retention rates
Study design	Randomized controlled trials (including pilot studies)

### Data collection

#### Selection of studies

All studies generated from the previously defined search strategies were evaluated against the pre‐defined inclusion criteria by two reviewers. Any disparities were addressed by involving a third reviewer and reaching an agreement. The studies that qualified for inclusion into the review were assessed with regards to their methodological quality by two reviewers. Studies were assigned a quality rating of low, high or unclear risk of bias for each criterion based on the Cochrane Collaboration's tool for assessing risk of bias 27. Studies were scored in relation to randomisation, allocation concealment, reporting of blinding, incomplete outcome data, selective outcome reporting and any other sources of bias (Supplementary materials Table S1). The two reviewers showed high inter‐rater reliability, and a third reviewer was not required (kappa = 0.89).

### Data extraction, synthesis and analysis

#### Primary outcome analysis

Weight loss was analysed at 3, 6 and 12 (or more) month data collection points as well as for the end of each study intervention.

#### Secondary outcome analysis

Outcomes of 5% weight loss, BMI change and waist circumference change were analysed at 3, 6 and 12 (or more) month data collection points as well as the end of each study intervention.

Retention rates are number of participants remaining and adhering to the randomized arm and also number of participants remaining in study for data collection (comparison with rates in the control group).

Coding of the BCTs was conducted for each of the studies, with 20% independently checked by the second reviewer. These were coded based on CALO‐RE taxonomy of BCTs to help people change their eating and physical activity behaviours 29. When coding for the presence of BCTs within an intervention, no assumptions were made. The standardized vocabulary within the BCT taxonomy was adhered to in order to state the presence of any BCT, explicitly or implicitly, within the interventions reported in each included paper, thus promoting consistent reporting and coding between researchers 19.

#### Analysis

Statistical analysis of the data was carried out using Review Manager 5. Data were analysed using mean (SD) change for each IWLPF and control group receiving no personalized feedback and compared whether significant differences were present between the different arms for each outcome measure: weight loss, BMI, waist circumference and 5% weight loss. Meta‐analysis was conducted to examine the studies at the end of each study intervention. As intervention length varied between the studies, time points were examined separately, including 3, 6 and 12‐month analysis in addition to the end of intervention. Meta‐analysis was conducted, with intention‐to‐treat analysis data if available from the published data, along with tests for heterogeneity. All study data included in the meta‐analysis used results measured at the end of the intervention. One of the included studies, by van Wier 30, conducted a follow‐up at 24 months (after a 6‐month intervention). Therefore, only post intervention data was used within the meta‐analysis. The follow‐up data of this study, 24 month, was not included to avoid the conflation of active loss and maintenance stage results. As a variety of control groups were included in the review, e.g. wait list, face‐to‐face and internet‐delivered, subgroup analyses were performed to separate the effect of feedback from that of delivery mode. Control groups were categorized into ‘waiting list or minimal face‐to‐face interventions’ and ‘control internet‐delivered interventions without personalized feedback’, refer to Table 2.

**Table 2:** Study descriptions

Study	Intervention group descriptions	Feedback type provided	Control group descriptions	Control group category
Appel 37	In person – a lifestyle intervention: 9 introductory modules and 21 additional modules	Computer‐generated	Control – self‐directed weight loss	Wait list/minimal face‐to‐face intervention
	Remote support – same features as the ‘in person’ intervention previously mentioned but delivered remotely, via an internet website	Computer‐generated		
Chambliss 38	Basic – received individualized tailored calorie plan and instructions on use of the weight management software system	Human delivered	Waiting list control	Wait list/minimal face‐to‐face intervention
	Enhanced – same as basic intervention but also included behavioural weight management strategies	Human delivered		
Collins 31	Basic – web‐based nutritional and exercise program	Computer‐generated	Waiting list control	Wait list/minimal face‐to‐face intervention
	Enhanced – same as previously mentioned but added automated personalized feedback	Computer‐generated		
Hunter 32	Behavioural internet therapy (BIT) plus usual care, asked to restrict calories and increase PA. LEARN programme, behavioural modification approach to weight management. Personalized advice based on diet and physical activity inputted data, received online.	Human delivered	Usual care – refer to primary care provider for a preventive health visit	Wait list/minimal face‐to‐face intervention
Kraschnewski 35	Achieve together website implementing 36 weight control behaviours, e.g. eating healthy snacks, plan what you will eat, write down what you eat and drink	Computer‐generated	Waiting list control	Wait list/minimal face‐to‐face intervention
McConnon 36, 47	Intervention website: combination of dietary advice, PA advice and behaviour therapy	Computer‐generated	Usual approach to weight loss care and printed information	Wait list/minimal face‐to‐face intervention
Morgan 39	Workplace POWER program providing weight loss advice with a counsellor via a website	Human delivered	Wait list control	Wait list/minimal face‐to‐face intervention
Morgan 40, 48	SHED‐IT Internet group, weight loss workplace website with online counsellor sessions	Human delivered	One face‐to‐face information session, weight loss booklet but no website access	Wait list/minimal face‐to‐face intervention
Tate 34	Website access resources about diet and exercise with included individualized feedback	Human delivered	Internet behavioural therapy (IBT) – website access resources about diet and exercise	Control internet‐delivered intervention receiving no personal feedback
Tate 22	Website providing a tutorial on weight loss with communication with weight loss counsellor	Human delivered	Basic internet group – website providing a tutorial on weight loss	Control internet‐delivered intervention receiving no personal feedback
Tate 33	Automatic counselling – use of Slim Fast website and pre‐programmed computer feedback	Computer‐generated	No counselling – use of Slim Fast website, weekly reports on diet intake, physical activity and weight loss	Control internet‐delivered intervention receiving no personal feedback
	Human counselling – use of Slim Fast website and feedback via human weight loss counsellor	Human delivered		
Van Wier 30	Phone – lifestyle intervention workbook and consultations provided via telephone with their counsellor	Human delivered	Self‐help brochures about overweight, healthy diet and PA	Wait list/minimal face‐to‐face intervention
	Internet – workbook accessed through an interactive website and contacted by their counsellor via the website	Human delivered		

## Results

**Figure 1:**
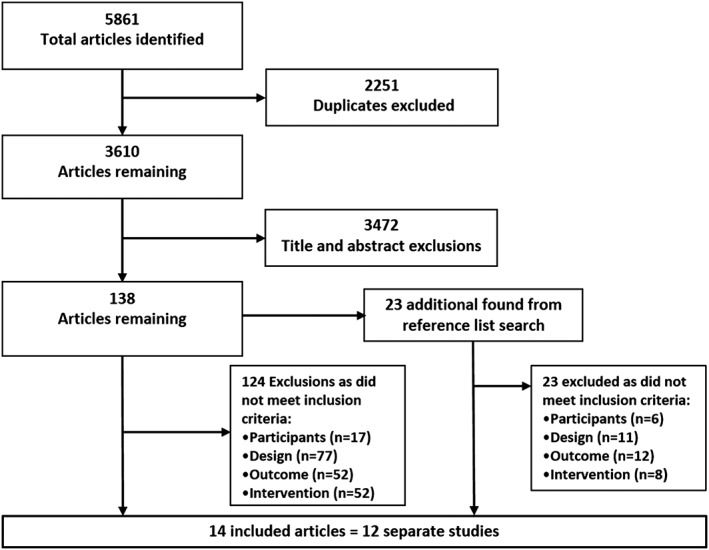
Screening for eligible studies.

Fourteen articles reporting on 12 separate studies were included in the review (Fig. 1).

Study quality assessment identified that only two of the studies assessed received low risk of bias for all criteria. All quality assessments can be found in Supplementary material Table S1. Selective reporting was the only criterion to receive high risk of bias scores for four of the studies 22, 30, 31, 32. Three studies provided monetary incentives for the completion of assessments that may have acted as a co‐intervention in respect of retention rates 22, 33, 34.

### Description of included studies

The characteristics of the included studies are summarized in Tables 2 and 3. All studies took place between 2001 and 2012. The majority (seven) were conducted in the USA, three in Australia, one in the Netherlands and one in the UK. The total number of participants was 3547 with 1816 females (51.2%). All 12 studies targeted changes to physical activity and diet. The length of the active interventions ranged from 3 to 24 months (21‐month range, mean 8.4, SD 5.7). Seven studies included two arms, and five studies included three arms. The studies varied in terms of the features of control/comparison arms (Table 2).

**Table 3:** Study recruitment, retention and intervention length

Study	Setting	*N*	*N* per arm	Percentage of females	Retention	Intervention length	Follow‐up
Appel 2011 37	USA	415	A) 138 B) 138 C) 139	264/415 (63.6%)	394/415 (94.9%)	24 months	None
Chambliss 38	USA	120	A) 30 B) 45 C) 45	99/120 (83%)	95/120 (79.2%)	3 months	None
Collins 31	Australia	309	A) 104 B) 99 C) 106	180/309 (58%)	260/30 (84.1%)	3 months	None
Hunter 32	USA	446	A) 222 B) 224	224/446 (50%)	399/446 (89.5%)	6 months	None
Kraschnewski 2011 35	USA	100	A) 50 B) 50	69/100 (69%)	88/100 (88%)	3 months	None
McConnon 2007/2009 36, 47	UK	221	110 B) 111	170/221 (77%)	131/221 (59.3%)	12 months	None
Morgan 2011a 39	Australia	110	A) 45 B) 65	All male (0%)	90/110 (81.8%)	3 months	14 weeks
Morgan 2011b 40, 48	Australia	65	A) 31 B) 34	All male (0%)	46/65 (70.8%)	12 months	None
Tate 2001 34	USA	91	A) 45 B) 46	81/91 (89%)	71/91 (78%)	6 months	None
Tate 2003 22	USA	92	A) 46 B) 46	83/92 (90%)	77/92 (83.7%)	12 months	None
Tate 2006 33	USA	192	A) 67 B) 61 C) 64	162/192 (84.3%)	155/192 (80.7%)	6 months	None
Van Wier 2011 30	Netherlands	1386	A) 448 B) 453 C) 450	457/1386 (33%)	792/1386 (57.1%)	6 months	24 months

## Provision of individualized feedback

Across the 12 studies, 8 incorporated human‐delivered internet feedback and 5 provided computer‐generated internet feedback (Table 2). One study provided the personalized feedback using both formats as the study contained two internet‐delivered intervention groups 33. These two terms have been used to distinguish between interventions using personalized feedback provided by a health care professional or researcher (human‐delivered) in contrast to personalized feedback created using algorithms to send pre‐programmed responses based on participant input or choices (computer‐generated). All 12 studies used personalized feedback to target information received on participant's weight loss progress or individual behaviour change, such as diet or physical activity level. Participant access to the internet‐delivered personalized feedback was via the website (four studies) or via emails containing the feedback (six studies), with two studies remaining unclear in how it was administered. Frequency of feedback varied, the majority of studies (seven) providing it on a weekly basis. In addition to personalized feedback, two studies sent computer‐generated messages when participants logged into the website 35, 36. One study provided computer‐generated messages to participants on completion of lesson modules or assessments 30.

### Meta‐analysis/synthesis of results

#### Internet weight loss interventions providing personalized feedback versus control groups receiving no personalized feedback

**Figure 2:**
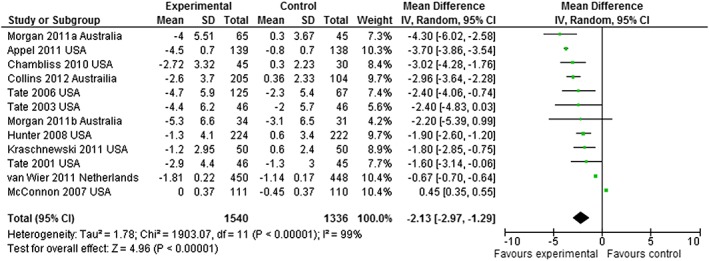
Forest plot weight loss results.

The primary outcome, weight loss, is shown in Fig. 2 illustrating the meta‐analysis forest plot for the 12 studies. Meta‐analysis identified that provision of feedback resulted in 2.13 kg (mean difference [MD]) (*p* < 0.00001) greater weight loss for the IWLPF in comparison with control groups receiving no personalized feedback. Heterogeneity levels showed considerable and significant heterogeneity (*I* ^2^ = 99%, *p* < 0.001) between control groups not receiving personalized feedback and the IWLPF. All outcomes were found to be statistically and clinically (≥5% body weight loss) significant for study end of intervention results (Table 4). This was also true for results from data collection conducted at 3 and 6 months. In contrast, studies with duration 12 months or over did not identify significantly greater weight loss for the IWLPF compared with control groups receiving no personalized feedback. A higher proportion of intervention participants reached ≥5% weight loss, but this was not significantly different at ≥12 months (1.53 [0.82, 2.84]; *p* = 0.18). Only BMI and waist circumference outcomes illustrated statistically significantly greater losses for the IWLPF compared with the control groups receiving no personalized feedback ≥12 months. All meta‐analysis forest plots can be found as supplementary material (Supplementary materials Figures S1–S4).

**Table 4:** Intervention versus control group meta‐analysis results at each data collection point

Time (months)	*N*	Weight loss	*N*	5% Weight loss (risk ratio)	*N*	BMI change	*N*	Waist circumference
End of intervention	12	−2.13 [−2.97, −1.29]*	10	2.13 [1.56, 2.90]*	8	−0.99 [−1.28, −0.70]*	8	−2.42 [−3.65, −1.19]***
3	7	−2.62 [−3.14, −2.09]*	3	8.26 [3.24, 21.07]*	5	−1.02 [−1.23, −0.81]*	5	−2.39 [−4.67, −0.11]***
6	7	−1.82 [−3.32, −0.32]***	5	2.30 [1.49, 3.55]***	3	−0.95 [−1.79, −0.11]***	4	−2.35 [−3.95, −0.76]***
≥12	4	−2.18 [−5.80, −1.44]	2	1.53 [0.82, 2.84]	3	−1.20 [−1.74, −0.66]**	2	−2.44 [−4.45, −0.42]***

Mean difference [95% CI].

* *p* < 0.00001.

** *p* < 0.0001.

*** *p* < 0.05.

*N*, number of studies included in meta‐analysis.

Retention rates were calculated by the number of participants who provided follow‐up data at the last assessment point (varying between studies). In total, intervention groups retained 73.5% (1132/1540) of participants, whilst control groups retained 77.5% (1036/1336), a significant difference in retention rates between intervention and control groups (*p* < 0.05).

#### Subgroup analysis

In nine studies, the control groups not receiving personalized feedback took the form of wait list or minimal face‐to‐face interventions 30, 31, 32, 35, 36, 37, 38, 39, 40. Minimal interventions included one‐off usual care appointments where participants received standardized weight loss‐printed information. Meta‐analysis showed a statistically significantly greater weight loss (2.14 kg MD, *p* < 0.001) for those in the IWLPF in comparison with the wait list or minimal interventions. Heterogeneity was considerable and significant between the wait list or minimal control groups and intervention groups (*I* ^2^ = 100%, *p* < 0.00001) (Supplementary Figure S5).

Meta‐analysis was performed for the three studies using control internet‐delivered interventions without personal feedback 22, 33, 34. Results showed 2.05 kg (*p* < 0.0001) greater weight loss for the IWLPF in comparison with the control internet‐delivered interventions receiving no personal feedback. Heterogeneity was not important and non‐significant between the control internet‐delivered interventions receiving no personal feedback and the IWLPF (*I* ^2^ = 0%, *p* > 0.05) (Supplementary Figure S6).

#### Behaviour change techniques

More BCTs were present within the IWLPF (median = 8, IQR = 6) in comparison with the control groups receiving no personalized feedback (median = 1, IQR = 3). Across the included 12 studies, IWLPF incorporated 25 different BCTs, out of the 40 BCTs outlined within the CALO‐RE taxonomy 29, while the control groups receiving no personalized feedback included only 17; 14/40 BCTs were not included in any study arms. The number of BCTs included in the IWLPF varied widely (4–19). The most effective studies 33, 37, 40 in terms of weight loss (kg) ranged from 7 to 14 BCTs and were not consistent in relation to included BCTs.

The BCTs incorporated most frequently are represented in Table 5 along with mean weight loss for each study's intervention and control group. The most prevalent BCT was ‘providing information on consequences in general’. This was the only BCT that was present in the majority of the control groups receiving no personalized feedback. Common techniques within the IWLPF, aside from ‘provide feedback on performance’, were ‘planning social support/social change’, ‘prompting self‐monitoring of behaviour/behavioural outcome’ and ‘goal setting (behaviour and outcome)’. These most commonly used BCTs tended to be clustered within the studies.

**Table 5:** Most commonly incorporated BCTs and effectiveness (mean weight change)

Study	Mean weight change	Info about consequences	Self‐monitoring (behaviour)	Feedback on performance	Goal setting (behaviour)	Social Support	Self‐monitoring (outcome)	Instruction on performing behaviour	Goal setting (outcome)	Barrier identification
Intervention groups										
Tate Human 33	−7.3	X	X	X	X	X	X	X		X
Morgan 40	−5.3	X	X	X		X	X		X	
Tate Automatic 33	−4.9	X	X	X	X	X	X	X		X
Appel 37	−4.5	X	X	X	X	X	X		X	X
Tate 22	−4.4	X	X	X						
Morgan 39	−4.0	X	X	X		X			X	X
Tate 34	−2.9	X	X	X		X	X	X		
Chambliss 38	−2.7	X	X	X	X					
Collins 31	−2.6	X	X	X	X	X	X	X	X	
Van Wier 30	−1.8	X	X	X	X					
Hunter 32	−1.3	X	X	X	X	X	X	X	X	X
Kraschnewski 35	−1.2	X	X	X	X			X	X	
McConn 36	0.0	X	X	X	X		X	X		
Trials using/not using		13/0	13/0	13/0	9/4	8/5	8/5	7/6	6/7	5/8
Control groups										
Morgan 40	−3.1	X								
Tate 33	−2.6	X	X				X	X		
Tate 22	−2.0	X	X							
Tate 34	−1.3	X								
Van Wier 30	−1.1	X								
Appel 37	−0.8	X			X	X			X	
McConn 36	−0.5	X								
Chambliss 38	0.3									
Morgan 39	0.3									
Collins 31	0.4									
Hunter 32	0.6	X						X		
Kraschnewski 35	0.6									
Trials using/not using		8/4	2/10	0/12	1/11	3/9	1/11	2/10	1/11	0/12

X = BCT present within the study group.

## Discussion

### Summary of key findings

Findings from this systematic review suggest that incorporating personalized feedback may be an important BCT for effective weight loss interventions delivered via the internet. Participants within the IWLPF were identified as twice more likely to achieve 5% weight loss than those in control groups. Shorter term data collection, 3 or 6 months, produced significant differences between the IWLPF and the control groups receiving no personalized feedback for all outcomes (weight loss, 5% weight loss, BMI and waist circumference change). In contrast, interventions lasting 12 months or longer did not produce significant differences between IWLPF and control groups receiving no personalized feedback for weight loss or 5% weight loss outcomes. Subgroup analysis identified significantly greater weight loss for the IWLPF irrespective of the comparator used, whether wait list/minimal face‐to‐face interventions or control internet‐delivered interventions receiving no personalized feedback.

### Comparison with previous literature

As in previous reviews, internet‐delivered weight loss interventions appeared to be more effective than comparison groups 13, 15. However, previously, in terms of significant differences between groups or clinical effectiveness of internet interventions, results were mixed 10, 11, 14, 16. The study by van Wier 30 conducting longer term follow‐up once the intervention had ended found similar findings to the results identified in this review. The significant difference between intervention and control groups identified after the intervention was delivered was lost by the 2‐year follow‐up.

Heterogeneity between included studies was evident and is a finding common in earlier reviews 41, 42, 43. Control group type appeared to impact on heterogeneity levels. Significant heterogeneity was identified between wait list/minimal face‐to‐face interventions and IWLPF. In contrast, heterogeneity levels between the control internet‐delivered interventions receiving no personalized feedback and the IWLPF were not significant, suggesting that the addition of feedback alone did not increase heterogeneity. Low heterogeneity suggests that feedback does not explain a great deal of the variability in interventions. The results from the BCT coding of study arms illustrated the variability between control groups receiving no personal feedback and IWLPF, with interventions containing more BCTs than the control groups. However, variability was also evident between the 12 IWLPF. The variability in included BCTs and weight loss achieved made it difficult to identify why particular studies were more effective. BCT coding identified that feedback was not the sole component that was commonly incorporated within the IWLPF. Instead, it appeared that the IWLPF used similar clusters of BCTs. However, one of these was self‐monitoring that is inherent to feedback in that participants would need to monitor their weight in order to gain feedback on it.

Attrition rates from previous reviews ranged from 20 to 43% 10, 13, 17, 44. Attrition rates in this review ranged from 12 to 47% and therefore are similar to previous findings. The review identified studies not reporting on several quality assessment criteria, with only two studies perceived low risk of bias for all criteria. Previous reviews also found mixed standards for reporting of quality criteria 11. This review identified the need for further improvement on the reporting of allocation concealment and blinding.

### Strengths and limitations of review

This review focused on personalized feedback in an attempt to explain differences in findings across the studies. It has illustrated how complex and variable internet weight loss interventions can be. A limitation of the review is the inability to control for all differences emerging from the different features, often leading to high heterogeneity levels identified and therefore makes comparison of internet‐delivered weight loss effectiveness very difficult to investigate. As a result, the influence of personalized feedback cannot be completely isolated from other intervention components. The BCTs used within the intervention groups were not consistent. Even within the most effective studies (in terms of weight loss), BCTs were incorporated differently. However, this approach highlights the need for researchers to both describe and investigate the exact content of interventions, to both improve replicability and to help isolate the effective components of interventions. The need to try to deconstruct complex interventions into their component elements to see what are the most effective ‘active ingredients’ is emphasized 45.

All the studies provided personalized feedback for weight loss or behaviour change (diet/physical activity). However, two studies generated messages when participants logged onto the websites 35, 36 and, one study 30, on the completion of modules or assessments. Both these participant interactions could have a potential effect on the intervention outcome; however, this does not appear to be the case with the three studies being placed in the four least effective studies when comparing mean weight loss difference between the intervention group and control group.

The lack of a set description when defining internet‐delivered weight loss intervention groups was a limitation with intervention names varied greatly, e.g. remote support, enhanced group or behavioural internet therapy. This was also a problem within the control groups, e.g. variability in the use of the term usual care. Following frameworks, such as TIDieR 46, may help to maintain a minimum standard when reporting intervention descriptions. Control groups tended to be wait list or usual care. Usual care allows real‐world practices to be examined in comparison to internet‐delivered weight loss interventions, but these were often what could be classified as minimal face‐to‐face interventions.

The majority of studies had high percentages of white, female participants, which could impact on the generalisability of the findings. Three studies provided monetary incentives for the completion of assessments, which may have biased the findings in terms of retention rates and thus outcome results 22, 33, 34.

### Implications for policy, practice and further research

Meta‐analysis results identified no significant weight loss for the IWLPF at longer term follow‐up (≥12 months). Long‐term maintenance is essential for health benefits, and therefore, more investigation is required to examine how weight loss could be maintained across time and how internet‐delivered interventions could be refined to better support weight maintenance. Further investigation into all BCTs used in each IWLPF and the relationship to effectiveness would be an important path to explore. Owing to small sample sizes within the included studies, analysing the relationship between effectiveness and BCTs could not be conducted in this review. This would be useful to examine in future research and would enable not only individual BCT impact to be investigated but also exploration of synergistic effects between clusters of BCTs and weight loss.

Human‐delivered internet feedback took the form of health care professionals or researchers producing individually created responses (emails/web‐based messages) to each participant, although the use of pre‐scripted responses for common queries/topics could be used. This causes potential limitations of scaling up an intervention as greater resources, labour and therefore costs would be incurred. This is especially true when compared against computer‐generated options available, which are less labour‐intensive after initial set‐up. However, human‐delivered internet feedback could still be more efficient in comparison with traditional face‐to‐face methods as there are wider issues such as the ability to provide health care advice quicker and easier because of greater flexibility, convenience and time efficiency for both health care professional and the patient. In addition, consultants have more readily accessible patient outcome data. Therefore, human‐delivered internet feedback is an important research area to investigate. One study 33 within this review compared internet feedback examining human‐delivered versus computer‐generated (with results favouring human‐delivered feedback), but research remains limited. Further research could highlight the advantages and disadvantages both options provide. Implications for practice relate to the use of IWLPF as alternative ways to provide weight management services. Further research is needed to establish whether internet‐delivered weight loss interventions provide additional benefit than in‐person services in current health care practice and to identify the most effective ways of providing personalized feedback.

## Conflict of interest statement

No conflict of interest was declared.

## Supporting information

Supporting info item

## References

[1] Health and Social Care Information Centre . Statistics for obesity, physical activity and diet: England. 2013.

[2] Office for National Statistics . Internet access: households and individuals. 2013.

[3] Internet Live Stats . Internet users by country. 2015; Available from: http://www.internetworldstats.com/stats.htm. Accessed 30th July 2015

[4] Jebb SA , Ahern AL , Olson AD *et al.* Primary care referral to a commercial provider for weight loss treatment versus standard care: a randomised controlled trial. Lancet 2011; 378: 1485–92. 21906798 10.1016/S0140-6736(11)61344-5 PMC3207352

[5] Jolly K , Lewis A , Beach J *et al.* Comparison of range of commercial or primary care led weight reduction programmes with minimal intervention control for weight loss in obesity: lighten up randomised controlled trial. BMJ 2011; 343: d6500. 22053315 10.1136/bmj.d6500 PMC3208022

[6] Dombrowski SU , Sniehotta FF , Johnston M *et al.* Optimizing acceptability and feasibility of an evidence‐based behavioral intervention for obese adults with obesity‐related co‐morbidities or additional risk factors for co‐morbidities: an open‐pilot intervention study in secondary care. Patient Educ Couns 2012; 87: 108–119. 21907528 10.1016/j.pec.2011.08.003

[7] Griffiths F , Lindenmeyer A , Powell J , Lowe P , Thorogood M . Why are health care interventions delivered over the internet? a systematic review of the published literature. J Med Internet Res 2006; 8: e10. 16867965 10.2196/jmir.8.2.e10 PMC1550698

[8] Ramadas A , Quek KF , Chan CKY , Oldenburg B . Web‐based interventions for the management of type 2 diabetes mellitus: a systematic review of recent evidence. Int J Med Inform 2011; 80: 389–405. 21481632 10.1016/j.ijmedinf.2011.02.002

[9] Broekhuizen K , Kroeze W , Poppel M , Oenema A , Brug J . A systematic review of randomized controlled trials on the effectiveness of computer‐tailored physical activity and dietary behavior promotion programs: an update. Ann Behav Med 2012; 44: 259–286. 22767052 10.1007/s12160-012-9384-3 PMC3442159

[10] Neve M , Morgan PJ , Jones PR , Collins CE . Effectiveness of web‐based interventions in achieving weight loss and weight loss maintenance in overweight and obese adults: a systematic review with meta‐analysis. Obes Revs 2010; 11: 306–321. 19754633 10.1111/j.1467-789X.2009.00646.x

[11] Norman GJ , Zabinski MF , Adams MA , Rosenberg DE , Yaroch AL , Atienza AA . A review of eHealth interventions for physical activity and dietary behavior change. Am J Prev Med 2007; 33: 336–345. 17888860 10.1016/j.amepre.2007.05.007 PMC2180189

[12] Neville LM , O'Hara B , Milat AJ . Computer‐tailored dietary behaviour change interventions: a systematic review. Health Educ Res 2009; 24: 699–720. 19286893 10.1093/her/cyp006 PMC2706490

[13] Arem H , Irwin M . A review of web‐based weight loss interventions in adults. Obes Revs 2011; 12: e236–e243. 20804523 10.1111/j.1467-789X.2010.00787.x PMC3086798

[14] Enwald KHP , Huotari AM‐L . Preventing the obesity epidemic by second generation tailored health communication: an interdisciplinary review. J Med Internet Res 2010; 12: e24. 20584698 10.2196/jmir.1409 PMC2956235

[15] Neville LM , Milat AJ , O'Hara B . Computer‐tailored weight reduction interventions targeting adults: a narrative systematic review. Health Prom J of Aus 2009; 20: 48–57. 10.1071/he09048 19402816

[16] Reed VA , Schifferdecker KE , Rezaee ME , O'Connor S , Larson RJ . The effect of computers for weight loss: a systematic review and meta‐analysis of randomized trials. J Gen Intern Med 2012; 27: 99–108. 21805218 10.1007/s11606-011-1803-9 PMC3250551

[17] Coons MJ , Demott A , Buscemi J *et al.* Technology interventions to curb obesity: a systematic review of the current literature. Curr Cardiovasc Risk Rep 2012; 6: 120–134. 23082235 10.1007/s12170-012-0222-8 PMC3471367

[18] Michie S , Richardson M , Johnston M *et al.* The behavior change technique taxonomy (v1) of 93 hierarchically clustered techniques: building an international consensus for the reporting of behavior change interventions. Ann Behav Med 2013; 46: 81–95. 23512568 10.1007/s12160-013-9486-6

[19] Abraham C , Michie S . A taxonomy of behavior change techniques used in interventions. Health Psychol 2008; 27: 379–387. 18624603 10.1037/0278-6133.27.3.379

[20] Khaylis A , Yiaslas T , Bergstrom J , Gore‐Felton C . A review of efficacious technology‐based weight‐loss interventions: five key components. Telemed J E Health 2010; 16: 931–8. 21091286 10.1089/tmj.2010.0065 PMC3000900

[21] Tang J , Abraham C , Greaves C , Yates T . Self‐directed interventions to promote weight loss: a systematic review of reviews. J Med Internet Res 2014; 16: e58. 24554464 10.2196/jmir.2857 PMC3961624

[22] Tate DF , Jackvony EH , Wing RR . Effects of Internet behavioral counseling on weight loss in adults at risk for type 2 diabetes – a randomized trial. JAMA 2003; 289: 1833–1836. 12684363 10.1001/jama.289.14.1833

[23] Krukowski RA , Harvey‐Berino J , Ashikaga T , Thomas CS , Micco N . Internet‐based weight control: the relationship between web features and weight loss. Telemed J E Health 2008; 14: 775–82. 18954247 10.1089/tmj.2007.0132 PMC2998280

[24] Carver CS , Scheier MF . Control theory: a useful conceptual framework for personality‐social, clinical, and health psychology. Psychol Bull 1982; 92: 111–135. 7134324

[25] Michie S , Abraham C , Whittington C , McAteer J , Gupta S . Effective techniques in healthy eating and physical activity interventions: a meta‐regression. Health Psychol 2009; 28: 690–701. 19916637 10.1037/a0016136

[26] Dombrowski SU , Sniehotta FF , Avenell A , Johnston M , MacLennan G , Araújo‐Soares V . Identifying active ingredients in complex behavioural interventions for obese adults with obesity‐related co‐morbidities or additional risk factors for co‐morbidities: a systematic review. Health Psychol Rev 2011; 6: 7–32.

[27] Higgins JPT , Green S . Cochrane Handbook for Systematic 3Reviews for Interventions Version 5.1.0 [updated March 2011], in The Cochrane Collaboration, 2011 Available from www.cochrane‐handbook.org. 2011.

[28] Moher D , Liberati A , Tetzlaff J , Altman DG , The PRISMA Group . Preferred Reporting Items for Systematic Reviews and Meta‐Analyses: The PRISMA Statement. PLoS Med 2009; 6: e1000097 DOI: 10.1371/journal.pmed1000097. 19621072 10.1371/journal.pmed.1000097 PMC2707599

[29] Michie S , Ashford S , Sniehotta FF , Dombrowski SU , Bishop A , French DP . A refined taxonomy of behaviour change techniques to help people change their physical activity and healthy eating behaviours: the CALO‐RE taxonomy. Psychol Health 2011; 26: 1479–1498. 21678185 10.1080/08870446.2010.540664

[30] Van Wier MF , Arins GAM , Dekkers JC , Hendriksen IJM , Smid T , Van Mechelen W . Phone and e‐mail counselling are effective for weight management in an overweight working population: a randomized controlled trial. BMC Public Health 2009; 9: 1–12. 19134171 10.1186/1471-2458-9-6 PMC2667416

[31] Collins CE , Morgan PJ , Jones P *et al.* A 12‐week commercial web‐based weight‐loss program for overweight and obese adults: randomized controlled trial comparing basic versus enhanced features. J Med Internet Res 2012; 14: e57. 22555246 10.2196/jmir.1980 PMC3376507

[32] Hunter CM , Peterson AL , Alvarez LM *et al.* Weight management using the internet: a randomized controlled trial. Am J Prev Med 2008; 34: 119–126. 18201641 10.1016/j.amepre.2007.09.026

[33] Tate DF , Jackvony EH , Wing RR . A randomized trial comparing human e‐mail counseling, computer‐automated tailored counseling, and no counseling in an Internet weight loss program. Arch Intern Med 2006; 166: 1620–5. 16908795 10.1001/archinte.166.15.1620

[34] Tate DF , Wing RR , Winett RA . Using Internet‐based technology to deliver a behavioral weight loss program. JAMA 2001; 285: 1172–1177. 11231746 10.1001/jama.285.9.1172

[35] Kraschnewski JL , Stuckey HL , Rovniak LS *et al.* Efficacy of a weight‐loss website based on positive deviance: a randomized trial. Am J Prev Med 2011; 41: 610–614. 22099238 10.1016/j.amepre.2011.08.012 PMC12456025

[36] McConnon Á , Kirk SFL , Cockroft JE *et al.* The Internet for weight control in an obese sample: results of a randomised controlled trial. BMC Health Serv Res 2007; 7. 10.1186/1472-6963-7-206 PMC2228294 18093289

[37] Appel LJ , Clark JM , Yeh HC *et al.* Comparative effectiveness of weight‐loss interventions in clinical practice. N Engl J Med 2011; 365: 1959–1968. 22085317 10.1056/NEJMoa1108660 PMC4074540

[38] Chambliss HO , Huber RC , Finley CE , McDoniel SO , Kitzman‐Ulrich H , Wilkinson WJ . Computerized self‐monitoring and technology‐assisted feedback for weight loss with and without an enhanced behavioral component. Patient Educ Couns 2011; 85: 375–382. 21295433 10.1016/j.pec.2010.12.024

[39] Morgan PJ , Collins CE , Plotnikoff RC *et al.* Efficacy of a workplace‐based weight loss program for overweight male shift workers: the Workplace POWER (Preventing Obesity Without Eating like a Rabbit) randomized controlled trial. Prev Med 2011; 52: 317–325. 21300083 10.1016/j.ypmed.2011.01.031

[40] Morgan PJ , Lubans DR , Collins CE , Warren JM , Callister R . 12‐month outcomes and process evaluation of the SHED‐IT RCT: an internet‐based weight loss program targeting men. Obesity 2011; 19: 142–51. 20523304 10.1038/oby.2010.119

[41] Manzoni GM , Pagnini F , Corti S , Molinari E , Castelnuovo G . Internet‐based behavioural interventions for obesity: an updated systematic review. Clin Pract Epi in Mental Health 2011; 7: 19–28. 10.2174/1745017901107010019 PMC3087973 21552423

[42] Shaw K , O'Rourke P , Del Mar C , Kenardy J . Psychological interventions for overweight or obesity. Cochrane Database Syst Rev 2005; 5: CD003818. 10.1002/14651858.CD003818.pub2 15846683

[43] Norris SL , Zhang X , Avenell A , Gregg E , Schmid CH , Lau J . Long‐term non‐pharmacological weight loss interventions for adults with prediabetes. Cochrane Database Sys Revs 2005; 2: CD005270. 10.1002/14651858.CD005270 PMC13356979 15846748

[44] Melville KM , Casey LM , Kavanagh DJ . Dropout from Internet‐based treatment for psychological disorders. Br J Clin Psychol 2010; 49: 455–471. 19799804 10.1348/014466509X472138

[45] Sutcliffe K , Thomas J , Stokes G , Hinds K , Bangpan M . Intervention Component Analysis (ICA): a pragmatic approach for identifying the critical features of complex interventions. Sys Rev 2015; 4: 1–13. 10.1186/s13643-015-0126-z PMC4627414 26514644

[46] Hoffmann TC , Glasziou PP , Boutron I *et al.* Better reporting of interventions: template for intervention description and replication (TIDieR) checklist and guide. BMJ 2014; 348: g1687. 24609605 10.1136/bmj.g1687

[47] McConnon A , Kirk SFL , Ransley JK . Process evaluation of an internet‐based resource for weight control: use and views of an obese sample. J Nutr Educ Beh 2009; 41: 261–267. 10.1016/j.jneb.2008.07.008 19508931

[48] Morgan PJ , Warren JM , Lubans DR , Collins CE , Callister R . Engaging men in weight loss: experiences of men who participated in the male only SHED‐IT pilot study. Obes Res Clin Prac 2011; 5: e239–e248. 10.1016/j.orcp.2011.03.002 24331106

