# Supplementary material for: Systematic review and meta‐analysis of internet‐delivered interventions providing personalized feedback for weight loss in overweight and obese adults
Source: Obes Rev. 2016 Mar 7;17(6):541–51. doi: 10.1111/obr.12396 (PMC4999041; doi:10.1111/obr.12396)

**Supplementary Materials**

**Systematic Review of internet-based interventions providing individualised feedback for weight loss in overweight and obese adults.**

Anna Sherrington^1 2 3^, James Newham^1^, Ruth Bell^1^, Ashley Adamson^1 2 3^, Elaine McColl^1 4^ and Vera Araujo-Soares^1^

^1^ *Institute of Health and Society, Newcastle University, Newcastle upon Tyne, NE2 4AX, UK*

*^2^ Human Nutrition Research Centre, Newcastle University, Newcastle upon Tyne, NE2 4HH, UK*

*^3^ Fuse–UKCRC Centre for Translational Research in Public Health, Newcastle University, Newcastle upon Tyne, NE2 4AX, UK*

^4^ *Newcastle Clinical Trials Unit, Newcastle University, Newcastle upon Tyne, NE2 4AE*

Corresponding author: Anna Sherrington - [anna.sherrington@newcastle.ac.uk](mailto:anna.sherrington@newcastle.ac.uk)

**Table S1: Study quality assessment**

| **Study** | **Randomisation** | **Allocation**  **concealment** | **Blinding** | **Incomplete outcome data** | **Selective outcome reporting** |
| --- | --- | --- | --- | --- | --- |
| Appel [[37](#_ENREF_37)] | + | ? | + | + | + |
| Chambliss [[38](#_ENREF_38)] | + | + | ? | + | ? |
| Collins [[31](#_ENREF_31)] | + | + | + | + | - |
| Hunter [[32](#_ENREF_32)] | + | ? | + | + | - |
| Kraschnewski [[35](#_ENREF_35)] | + | + | ? | + | ? |
| McConnon [[36](#_ENREF_36), [48](#_ENREF_48)] | + | + | + | + | + |
| Morgan a [[39](#_ENREF_39)] | + | + | ? | + | + |
| Morgan b [[40](#_ENREF_40), [49](#_ENREF_49)] | + | + | + | + | + |
| Tate 2001 [[34](#_ENREF_34)] | + | ? | ? | + | ? |
| Tate 2003 [[22](#_ENREF_22)] | + | ? | ? | + | - |
| Tate 2006 [[33](#_ENREF_33)] | + | ? | ? | ? | ? |
| Van Wier [[30](#_ENREF_30)] | + | v | + | ? | - |
| + = Low risk of bias, ? = Unclear risk of bias, - = High risk of bias | | | | | |

**Meta-analysis forest plots results**

**Figure S1: Internet feedback versus no feedback weight loss (kg)**

**
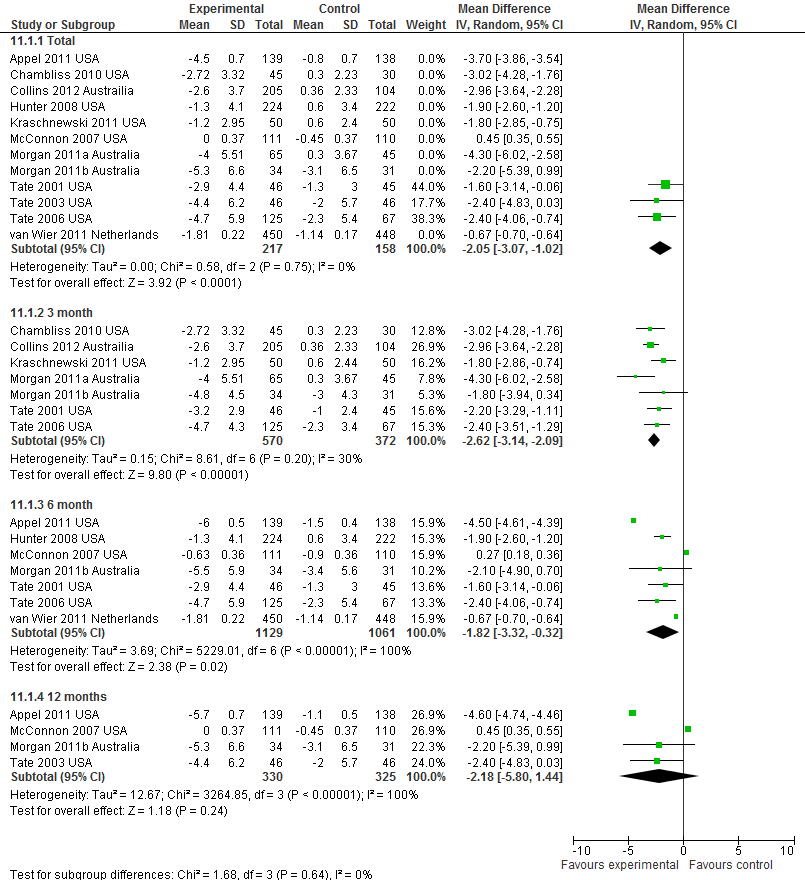
**

**Figure S2: Internet feedback versus no feedback 5% weight loss**


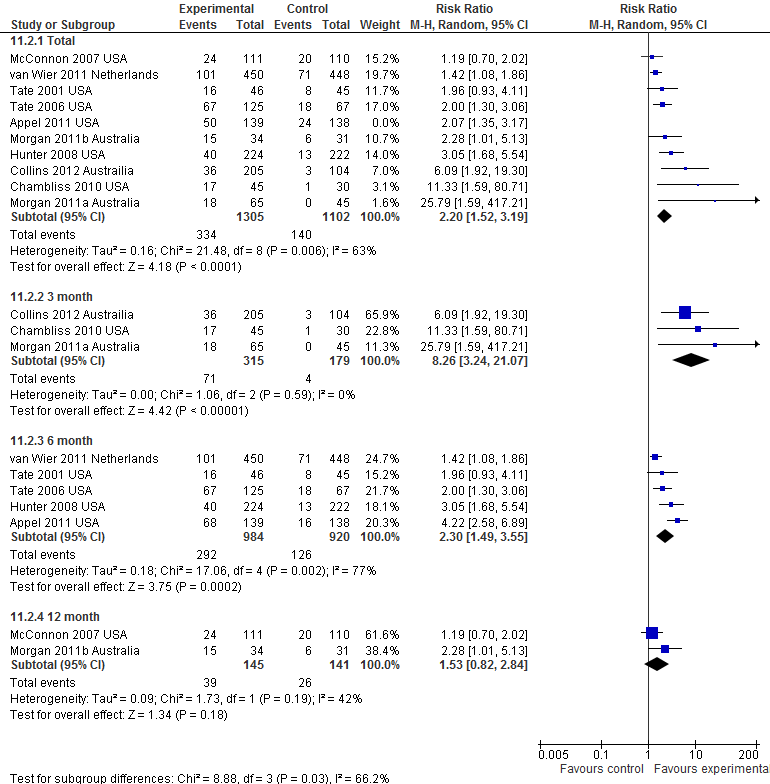


**Figure S3: Internet feedback versus no feedback mean waist circumference change**


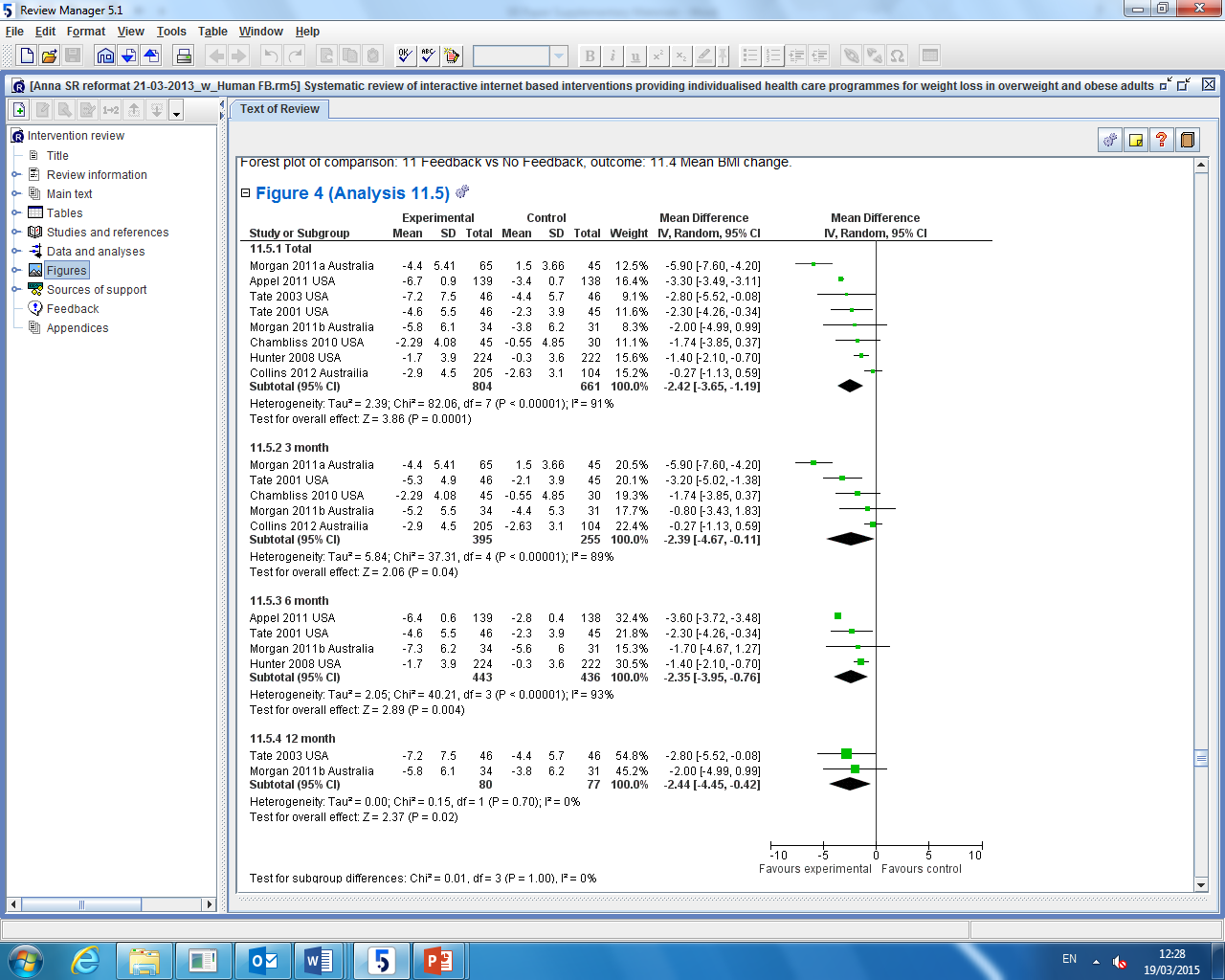


**Figure S4: Internet feedback versus no feedback mean BMI change**


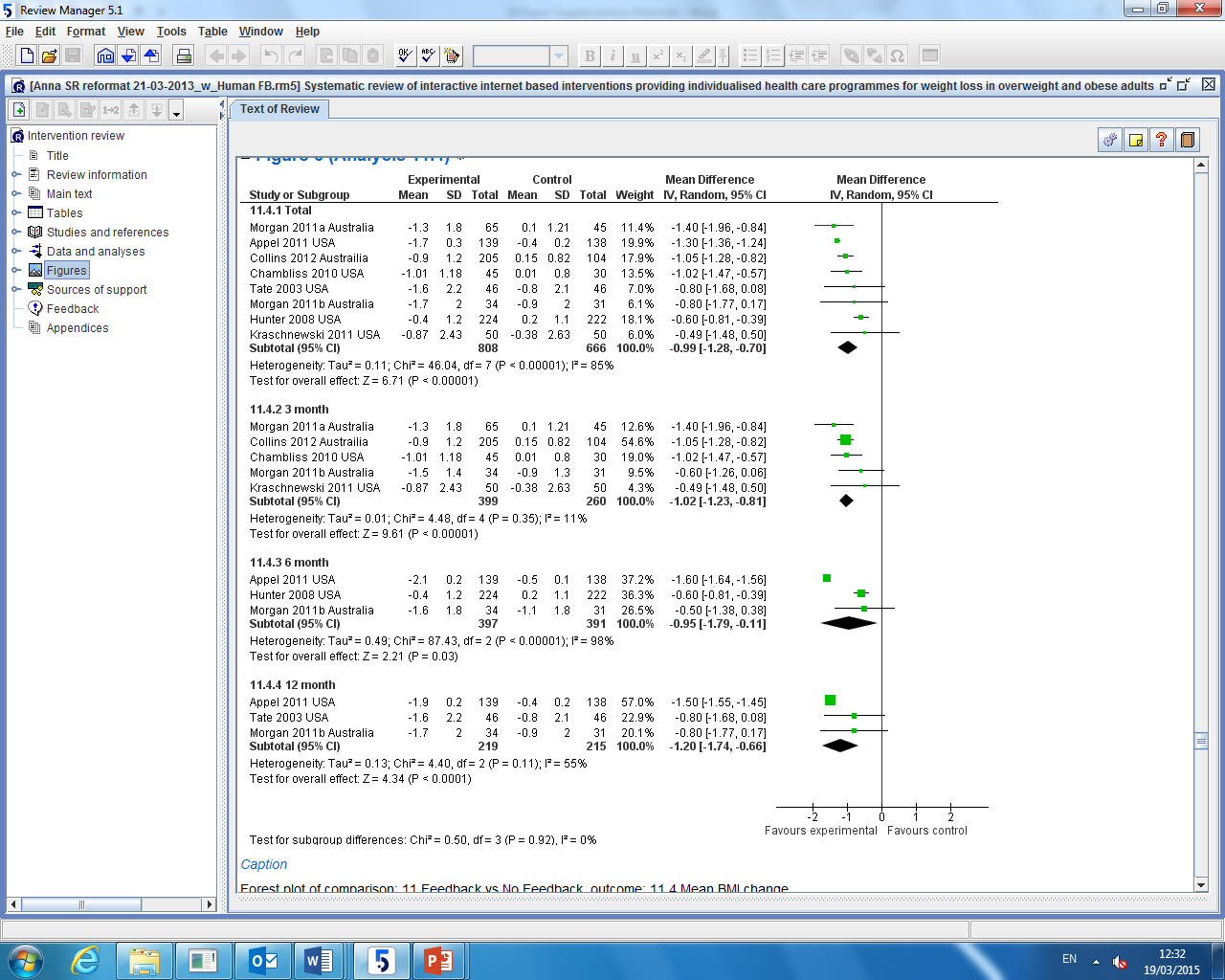


**Figure S5: Wait list control/minimal intervention versus internet feedback interventions mean weight loss (kg)**


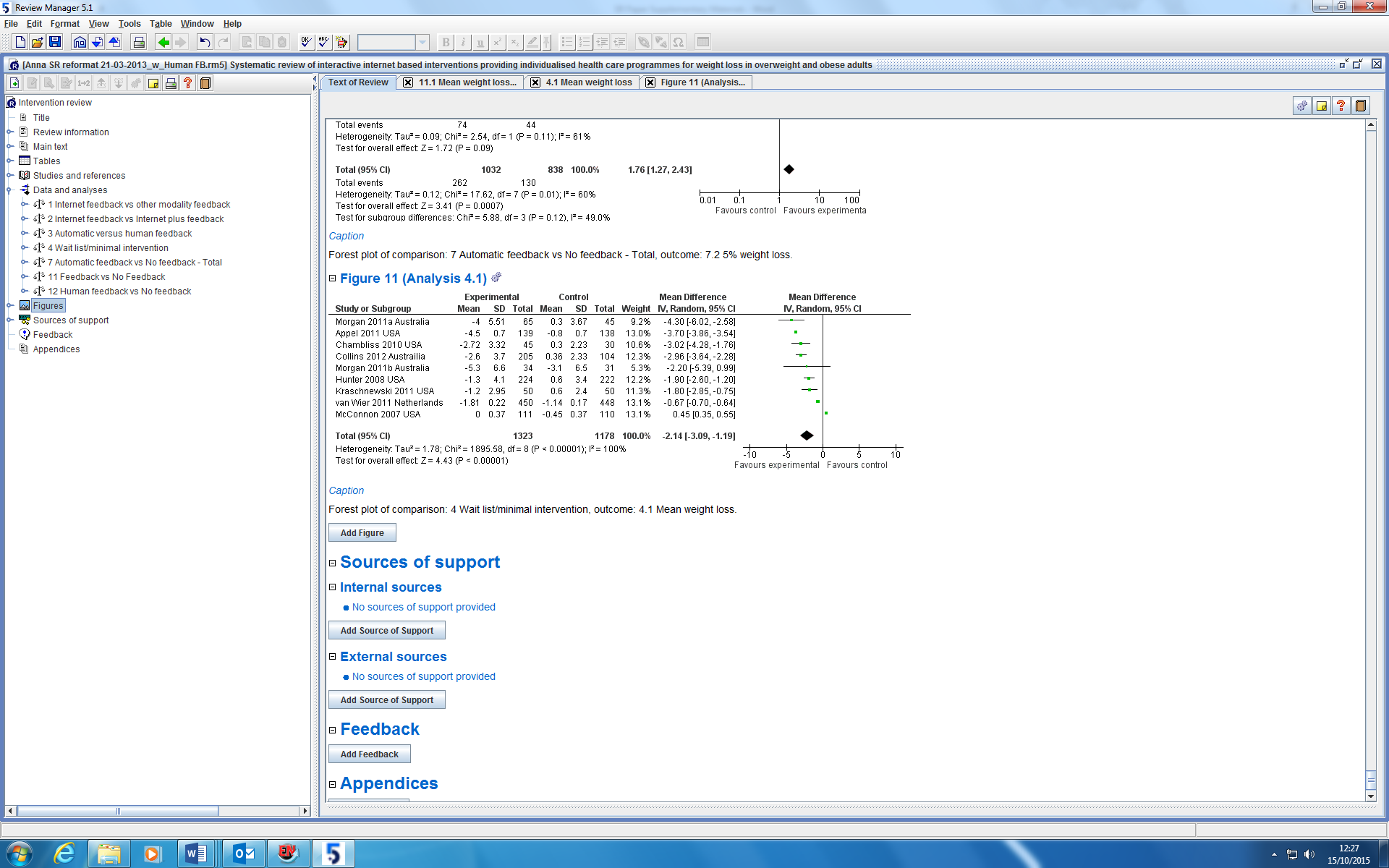


**Figure S6: Control interventions with no feedback versus internet feedback interventions mean weight loss (kg)**


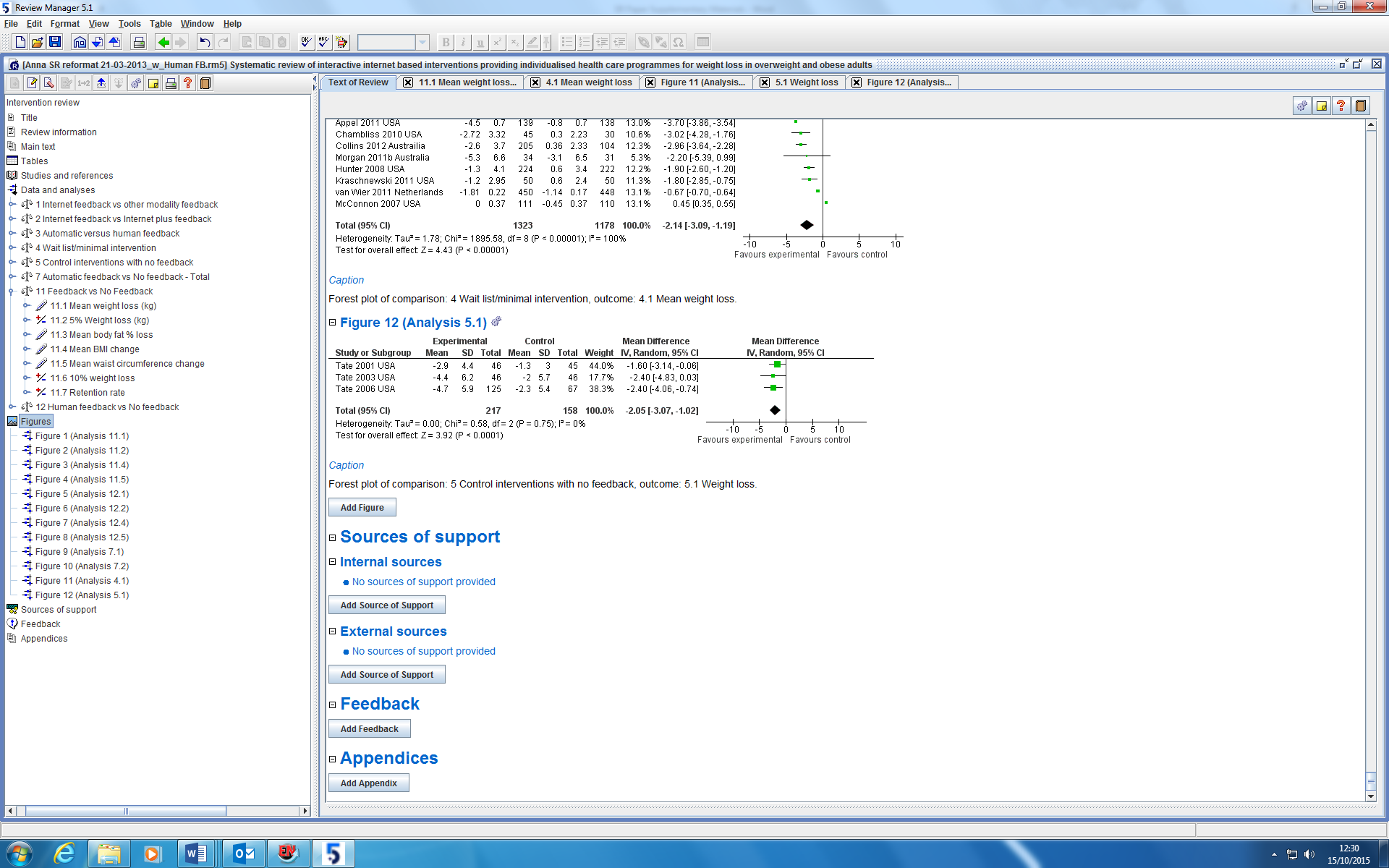

Supplement: Supplementary file 1 — Supporting info item [file OBR-17-541-s001.docx]
